# Clinical Application of Partial Splenic Embolization

**DOI:** 10.1155/2014/961345

**Published:** 2014-11-03

**Authors:** Yong-Song Guan, Ying Hu

**Affiliations:** ^1^Department of Oncology, West China Hospital, Sichuan University, Chengdu 610041, China; ^2^State Key Laboratory of Biotherapy, Sichuan University, Chengdu 610041, China

## Abstract

Partial splenic embolization (PSE) is one of the intra-arterial therapeutic approaches of diseases. With the development of interventional radiology, the applications of PSE in clinical practice are greatly extended, while various materials are developed for embolization use. Common indications of PSE include hypersplenism with portal hypertension, hereditary spherocytosis, thalassemia, autoimmune hemolytic anemia, splenic trauma, idiopathic thrombocytopenic purpura, splenic hemangioma, and liver cancer. It is also performed to exclude splenic artery aneurysms from the parent vessel lumen and prevent aneurysm rupture, to treat splenic artery steal syndrome and improve liver perfusion in liver transplant recipients, and to administer targeted treatment to areas of neoplastic disease in the splenic parenchyma. Indicators of the therapeutic effect evaluation of PSE comprise blood routine test, changes in hemodynamics and in splenic volume. Major complications of PSE include the pulmonary complications, severe infection, damages of renal and liver function, and portal vein thrombosis. The limitations of PSE exist mainly in the difficulties in selecting the arteries to embolize and in evaluating the embolized volume.

## 1. Introduction

Splenic embolization was first introduced in 1973, when autologous blood clot was used by Maddison [1] to produce splenic artery embolization for hypersplenism treatment. Seven years later, transcatheter partial splenic embolization (PSE) was developed by Spigos et al. [2], which has been proved as a safe and effective method of vascular occlusion. Since then, PSE has ever been gaining its indications and is popularly used in the world, nowadays, and increasingly performed to treat various clinical conditions from salvaging patients with blunt splenic injury [3] to facilitating interferon therapy in patients with chronic hepatitis virus infection [4].

## 2. The Mechanism of PSE

Hypersplenism, either primary or secondary, may enhance the progress of phagocytosis and the destruction of blood cells, and the enlarged spleen may retain a great quantity of blood cells inside the hyperplastic splenic sinusoids, which might result in a decrease in peripheral blood cells [5–7].

On the other hand, the spleen is a major source of antibodies and lymphocytes production and an enhancer of phagocytosis of white cells. It plays an important role in the immune system, too. Although splenectomy may eliminate the hypersplenism-caused blood cell destruction, it increases the risk of systemic infection outbreak as well and is prohibited in selected high-risk patients by both the surgical intervention and the resulting asplenic state [8]. Compared with PSE, splenectomy has some disadvantages such as higher invasiveness, longer hospital stay, increased blood loss, and more operative and postoperative complications [9]. More importantly, PSE helps to save splenic function [8].

PSE therefore was developed to achieve the occlusion of arteries in certain spleen areas and to overcome the limits of splenectomy [8]. PSE is a transcatheter embolization method, and successful embolization of the selected arteries results in devascularization of a focal lesion or in intentional reduction or cessation of blood flow into a target vascular bed or to the entire organ. The spleen is a perfect candidate for partial embolization because of its anatomical characters [10, 11]. When coming near the splenic hilum, the splenic artery usually divides into the superior and inferior terminal branch, and each branch further divides into four to six segmental intrasplenic branches. The spleen can be divided into many small segments on the basis of the distribution of its arterial blood supply, and there are few communicating branches between these different segments. Therefore, occlusion of a certain artery may affect only the corresponding area of blood supply, while function of the remaining part of the spleen could be reserved. However, extra- and intrasplenic anastomosis are still found in 6.6%–15.3% and 15.3%–43.3% in population, respectively, and anatomical variations [12] of splenic artery are also not uncommon, which may complicate the result and procedure of PSE. There is variation in origin, course, and terminal distribution pattern of the splenic artery. Sometimes, the proximal part of the splenic artery divides into two or more branches that have suprapancreatic and enteropancreatic courses [12]. The interventional radiologist must keep in his mind such variations.

## 3. Embolic Material for PSE

Many materials have been developed for embolization use, including stainless steel coils, gelatin sponge, silk suture, and autologous blood clot and polyvinyl alcohol (PVA). It was reported that there was no difference among the short-term therapeutic effects of stainless steel coils, gelatin sponge, and PVA embolization [8]. According to our practice, gelatin sponge [13], absolute alcohol [14], and lipiodol [15] are cheap materials readily available, used in easy-to-handle procedures.

Gelatin sponge is a popular material for embolization. When injected into an artery as small pieces, gelatin sponge will be rushed downstream until it can go no farther and blocks the vessel for a few days or up to 12 weeks. Hypersplenism can be relieved effectively by this material when the embolized area of spleen exceeds 50% [16]. However, the size of the gelatin sponge pieces is inappropriate for the embolization of the red pulp arteries, which makes it impossible for the embolization of splenic functional area [17]; the latter can be achieved successfully by silk suture floating which is a permanent embolic agent for proximal occlusion of vessels [18].

Stainless steel coils embolization [19] has less embolization pain or other complications, but with a higher relapse rate of hypersplenism. Platinum coil embolotherapy is also efficacious for occlusion of vessels, the same as that of stainless coil [20].

Embolization pain caused by PVA is earlier and more severe than by other materials. A reasonable explanation for this difference is that the infarction area of PVA embolization is located more closely to the periphery of the spleen, and the exudation which resulted from infarction should result in swelling even fluid accumulation under the adventitia of the spleen. However, as a permanent embolic material, PVA provides an embolization with less recanalization rate and lower incidence of fever than other materials do, which may be related to the fewer chances of fever and infection of PVA induced dry infraction of functional areas of the spleen [8, 19].

Super absorbent polymer microsphere (SAP-MS) [21] is another choice of embolic materials. It was reported that preoperative splenic artery embolization using SAP-MS would be effective for easy and safe laparoscopic or laparoscopically assisted splenectomy, and no major postoperative complications had been identified.

Cellulose sphere (CELPHERE, Asahi-Kasei Co., Ltd., Tokyo, Japan) is a FDA-approved biocompatible microcrystalline without expanding tendency when introduced into vessels, which is different to PVA beads or gelatin sponge beads. Kai et al. [22] proved that CELPHERE was a safe solid embolic material for permanent occlusion of blood vessels in an animal model. CELPHERE beads with various diameters traveled into vessels with approximating diameters of them. Twelve weeks after CELPHERE embolization, no evidence of vessel wall disruption, perivascular hemorrhage, or inflammatory changes was found. Till now, results to confirm the proper intravascular application of CELPHERE in human are still unavailable. Nevertheless, the beads are considered easy to handle and with few adverse effects and may be suitable for clinical application.

Xi et al. [23] reported the application of operative suture as embolization material for red pulp supplying artery embolization. 4- or 5-0 silk sutures were prepared in 2 mm sizes and were injected into the splenic artery through a catheter. The suture segments therefore floated downstream and blocked the red pulp supplying artery successfully.

## 4. Techniques of Splenic Embolization

### 4.1. Embolization of the Trunk of Splenic Artery

**Figure 1:**
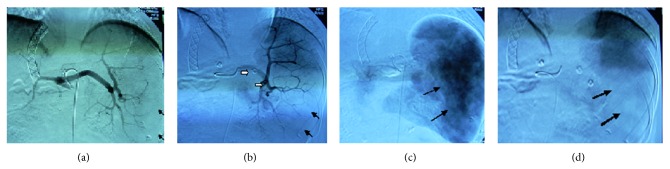
Severe cirrhotic liver has been managed with TIPS for relief of portal hypertension. (a) Angiogram shows the small size of cirrhotic liver and the enlarged spleen with abundant peripheral small arteries (arrows) before PSE. (b) The trunk and the lower splenic polar artery have been occluded by stainless steel coils (bold arrows). Arterial phase shows fewer peripheral small arteries in the lower part after embolization. (c) Venous phase of the angiogram before PSE shows the lower part with rich venous blood flow (arrows). (d) Compared with the upper part, the lower part after PSE has very poor venous flow (arrows).

Stainless steel spring coils or detachable balloons placed in a main branch of the splenic artery would temporally reduce the portal vein pressure and prevent the esophageal varices bleeding, with basically the same effects of ligation of the splenic artery [24]. This technique may also reduce the risk of surgery by improving the platelet counts before splenectomy. PSE by this technique may not cause wide-ranging infarction of the spleen, but hypersplenism may relapse after the development of collateral circulation. In severe cirrhotic cases, we often embolize both the trunk and the lower polar splenic artery (Figure 1).

### 4.2. PSE

The techniques of PSE reported vary greatly. Basically, they can be classified into two categories, including nonselective and selective catheterization methods. A new percutaneous technique has been described in which a narrowed stent is deployed to the middle portion of the splenic artery and is associated with lower frequencies of postintervention fever and pain, shorter hospital stay, and decreased need for antibiotics compared with PSE [25].

*(1) Low Pressure Flow Control Protocol.* Low pressure flow control protocol, by our experiences, is a nonselective method, by which the embolization materials are injected into a main branch of splenic artery through catheter and float downstream until they can not go further. Although the procedure is easy to be handled, the evaluation of the embolized volume is difficult. On the other hand, the unintentional embolization of the pancreatic artery by small embolic particles is often unavoidable. Main coil and subselective embolization have similar outcomes, and common complications do not seem to affect outcome [26].

**Figure 2:**
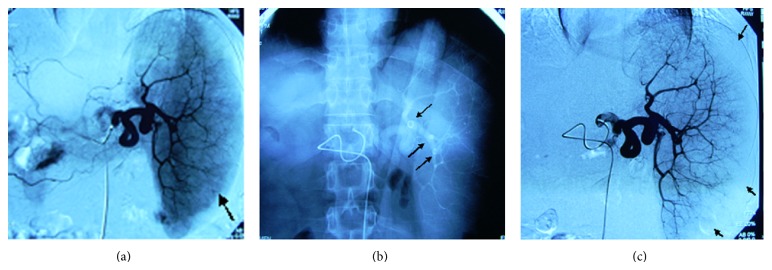
Embolization of the upper and lower polar arteries in combination with achieving peripheral infarction. (a) Arteriogram shows splenomegaly before PSE and abundant peripheral small arteries (arrow). (b) The upper and lower polar arteries have been embolized with coils (arrows). (c) Compared with (a), the peripheral area has scanty arterial branches (arrows).

*(2) Embolization of the Lower or Upper Polar Splenic Artery.* This is a technique with selective catheterization. The catheter tip is positioned in the main branch of the lower polar splenic artery. After an estimation of the volume to be embolized by observation with injecting contrast medium, embolization would be performed with permanently embolic materials. This method may minimize some complications related to PSE, such as atelectasis or pneumonia [2], and the symptoms caused by diaphragm or pleura irritation. In addition, the choice of selective catheterization may avoid the unintentional embolization of pancreatic artery. The lower and upper polar artery can be embolized in combination with achieving peripheral infarction of parenchymal tissue (Figure 2).

## 5. Indications of PSE

PSE was at first developed for primary and secondary hypersplenism [2]; however, following improvement of techniques, the indications have been greatly extended [4, 8].

### 5.1. Alteration of Blood Cell Count

PSE is proposed in patients with cirrhosis in cases when thrombocytopenia or neutropenia may cause clinical manifestations or if there are contraindications to other therapeutic procedures such as splenectomy [27]. PSE may resolve cytopenia, increasing red blood cell [16], white blood cell [27], and platelet count [4].

### 5.2. Cirrhosis with Portal Hypertension and Hypersplenism

Hypersplenism is a common condition in cirrhosis with portal hypertension [2, 8]. Recurrent bleeding would happen in more than 70% of portal hypertension patients with variceal bleeding history [18]. It is a general consensus that all patients who have previously bled from varices should be given further treatment to prevent rebleeding [14]. Decreased portal vein pressure is a major destination for this situation. Transjugular intrahepatic portal systemic shunt (TIPS) is one of the standard techniques for this aim; however, encephalopathy is encountered frequently [28].

Splenectomy is another effective treatment for hypersplenism; however, it also impairs the body's ability to confront microorganisms infection. On the other hand, patients after splenectomy may face more complex situations when a second surgical operation, such as liver transplantation, is needed [29].

Recently, PSE has been developed into an alternative choice for the treatment of portal hypertension and hypersplenism. Portal vein pressure is decreased after PSE since blood flow from the splenic artery is reduced. In addition, liver function was reportedly improved by PSE, with cholinesterase activated and level of serum albumin increased [30].

In a study involving thirty patients with portal hypertension and hypersplenism, PSE significantly improved the clinical manifestation of portal hypertensive gastropathy (splenic embolization versus control; 71% versus 8%), which implied the beneficial effect of PSE on portal hypertensive gastric mucosa in patients with hypersplenism [31].

### 5.3. Idiopathic Thrombocytopenic Purpura (ITP)

Approximately 50% of ITP patients responded to corticosteroids [32, 33]. However, there were patients who showed refractory to steroids or they did respond to steroids but could not be withdrawn from such agents; thus splenectomy was required as a consequence. Since the well-recognized limits of surgical treatment, PSE was used as an alternative technique. Miyazaki et al. [34] showed that either complete response (platelet greater than 100,000/mm^3^) or partial response (platelet greater than 50,000/mm^3^) was found in more than 70% of patients who were not sensitive to steroids. In those who later underwent splenectomy, response to PSE or splenectomy was coincident.

### 5.4. Hereditary Spherocytosis

Hereditary spherocytosis is a genetic disorder of red blood cell membrane and results in that the red cells being smaller, rounder, more fragile, and less flexible than normal. The spherocytes tend to get trapped in the spleen and are broken up there. Hereditary spherocytosis often shows up in infancy or early childhood, and the traditional treatment of hereditary spherocytosis is splenectomy. However, young children without a spleen are at increased risk for infection. Therefore, PSE was used as another potential option and was proven a safe and effective alternative to splenectomy [35].

### 5.5. Thalassemia

Thalassemia is a complex contingent of genetic disorders all of which involve underproduction of hemoglobin, the indispensable molecule in red blood cells that carries oxygen. In an earlier small sample trial, Pringle et al. [36] reported that after PSE, patients with beta-thalassemia major showed a marked reduction in transfusion requirements, which implied that PSE may provide an acceptable alternative to splenectomy in these patients with the possible preservation of some splenic immune function.

### 5.6. Autoimmune Hemolytic Anemia

Autoimmune hemolytic anemia is a condition in which the immune system attacks the red blood cells. The red blood cells are covered by low-order antibody, which may lead to cell membrane destruction and therefore develop to spherocytes. Since the spleen is the major location of the destruction of spherocytes, though PSE can not overcome the immune system disorder, the broken up of red blood cells should be decreased after embolization [37].

### 5.7. Splenic Trauma

Splenic trauma is a common consequence in blunt abdominal trauma. The treatment of splenic trauma depends on the clinical condition. Splenectomy, which is the traditional surgical management of splenic trauma, has been found related to the risk of fatal postsplenectomy sepsis and impaired resistance to certain infections later in life. Bed rest and observation has been a nonsurgical management choice for splenic trauma, with high failure rates especially in patients older than 55 years of age [38]. PSE has been reported to improve the results of nonsurgical treatment [39]. The most widely accepted indication of PSE in this situation is evidence of arterial injury on CT scans [3]. Embolization should be performed with microcoils in distal arterial branch supplying the segment on which extravasation, pseudoaneurysm, or abrupt termination is depicted. Although PSE is reported safe and associated with fewer complications for splenic injury as compared with surgical treatment, operation may be more suitable for the patients with higher injury severity scores, lower blood pressure, lower pH value, and increased number of packed red blood cell transfusions [39].

### 5.8. Splenic Hemangioma

Hemangiomas involving the spleen are rare and seldom symptomatic, but there is a high incidence of splenic hemangioma after liver transplantation, while the spontaneous rupture is a rare but mortal complication [40, 41]. Traditionally, bipolar surgical ligation of the splenic artery, ligation of the aneurysm, or aneurysmectomy with or without splenectomy was the life-saving and mandatory procedures for splenic hemangioma, with a mortality rate of approximately 1%. Recently, those surgical treatments have been replaced by PSE to some extent, since PSE is associated with significantly lower morbidity and mortality than the former [42].

### 5.9. Liver Cancer

Hepatic artery and portal vein provide most of the blood supply of liver cancer. PSE would greatly reduce the blood flow of portal vein, which may reduce the blood supply of cancer. The more important fact is that the portal venous pressure should be decreased up to 17% after PSE, which may reduce the risk of esophageal varices bleeding after TACE [43].

### 5.10. Pretreatment for Antiviral Therapy in Hepatitis Virus Infection

PSE is now used in a combination modality as a pretreatment to reduce cytopenia in hepatitis C virus-induced cirrhosis patients with hypersplenism, making antiviral therapy possible per se at higher dosages with a sustained duration [4].

### 5.11. Other Indications

It is also performed to exclude splenic artery aneurysms from the parent vessel lumen and prevent aneurysm rupture, to treat splenic artery steal syndrome and improve liver perfusion in liver transplant recipients, and to administer targeted treatment to areas of neoplastic disease in the splenic parenchyma [8].

## 6. Contraindications of PSE

The contraindications of PSE include secondary splenomegaly and hypersplenism with the original disease in terminal stage; pyemia; or other severe infections which implies high risk of splenic abscess after the procedure. For patients with prothrombin time less than 70% of normal control, a remedy treatment is necessary before PSE [44].

## 7. Indicators of Therapeutic Effects Evaluation

### 7.1. Platelet Count

A rise in platelet count would be found 12 to 24 h after PSE, while the peak count (beyond the lower limit of normal level) will be reached in 1 or 2 weeks [45]. The platelet count should be stable in 2 months at about 2 times higher than that before PSE, while the stable level may show a slight descent from peak. There is a positive correlation between the platelet count and the volume of spleen infarction. Platelets have been reported remaining significantly more numerous than before embolization for up to 8 years [16].

### 7.2. White Blood Cell Count

Transient elevation of the serum white blood cell count (WBC) is normal physiological responses after splenectomy, which could be found at day 1 postoperative and may reach their peak values at day 3 postoperative. However, a prolonged elevation of WBC may imply an infection. After PSE, leucocyte counts also increase markedly, 51% at 1 month and 30% at 6 months [27].

### 7.3. Erythrocyte

Erythrocyte destruction occurs almost exclusively in the enlarged spleen in cases of predominant splenomegaly [6]. Erythrocytes are destroyed in the splenic erythrocyte pool at a constant rate. One study [5] estimated quantitatively the splenic erythrocyte destruction rate to be between 0.5 and 4.4% of the total erythrocyte mass per day, increasing significantly with increasing splenic weight. A rise in the erythrocyte count may be found 3 months after PSE, which could be detected significantly increased at 6 months after the procedure even up to normal level, remaining increased for up to 7.5 years [16].

### 7.4. Alteration of Hemodynamics

Portal flow velocity was decreased in cirrhotic patients with Child's C cirrhosis, as compared to those with Child's A cirrhosis. Decrease in blood flow and increased congestion indexes in the portal vein and splenic vein are related to the impairment of liver function in cirrhotic patients [46]. PSE improves the local hyperdynamic state in the splenic area and damages neither the portal blood flow volume nor the liver function. It is safe and effective for hypersplenism improvement from the portal hemodynamic point of view [47].

### 7.5. Alteration of Splenic Volume

Fifty percent to 80% of spleen is usually devascularized by embolization, and the nonembolized volume can be used to predict the functional outcome of PSE [48]. Method of reduced-volume embolization, that is, embolization of 30%–40% of the splenic volume, has been used to decrease morbidity while platelet count is maintained above baseline and bleeding tendency is controlled. The splenic volume will reduce gradually in the following months after embolization. Care must be taken when splenic necrosis is over 70% after PSE, as subsequent splenic abscess may lead to death [27]. Contrast-enhanced CT scan is a precise technique for the measurement of the area of splenic infarction [49].

### 7.6. Improvement in Condition of Gastric Mucosa

It has been reported that PSE has a beneficial effect on portal hypertensive gastric mucosa in patients with hypersplenism [31]. Ohmagari et al. [31] found that PSE induced a 11% reduction in gastric mucosal hemoglobin content (*P* < 0.01) when assessed by reflectance spectrophotometry, while portal hypertensive gastropathy in PSE group was significantly improved versus control.

Other parameters are found that neither aspartate aminotransferase nor alanine aminotransferase activities in serum changed significantly during follow-up. Choline esterase activity increased significantly by 6 months after embolization and remained increased for more than 7 years. Serum albumin concentration increased significantly, beginning at 6 months after embolization; this increase was maintained for 6 years [16].

Alterations in results of these indicators after PSE are clinically significant. PSE significantly reduces the cytopenia induced by hypersplenism, especially thrombocytopenia, which impairs antiviral therapy of hepatitis virus infection by pegylated interferon and ribavirin as haematological toxicity [4]. PSE improves hematologic parameters in those who otherwise would be unable to undergo high-dose chemotherapy or immunosuppressive therapy [8]. PSE is also reported to improve hepatic function as a beneficial nonsurgical treatment that enhances hepatic protein synthetic capacity as well as alleviating hypersplenism [16].

## 8. Complications

### 8.1. Postembolization Syndrome

Postembolization syndrome is common and has been reported as 30% but generally resolving without sequelae [50]. It includes daily intermittent fever below 39°C, abdominal pain, nausea and vomiting, abdominal fullness, and appetite loss [51]. Abdominal pain and fever have been reported as 82% and 94%, respectively. The daily intermittent fever is usually related to the release of pyrogens by the inflammatory cells inside the infarcted area and requests for nominal therapy. However, a fever lasts for more than 7 days and beyond 39°C may imply the existence of infection. Abdominal pain is caused by the acute edema in the infarct area and should be alleviated in 3 to 10 days after effective pain treatment. The particle size of embolic material was reported as a major influence factor for the incidence of postembolization syndrome. It was found that embolic material in smaller size caused abdominal pain severer and earlier, but with less fever, and vice versa [25, 52].

### 8.2. Pulmonary Complications

Pulmonary complications of PSE include pneumonia, atelectasis, and pleural effusion [2]. These complications are usually found present in the left side of the body and after the upper pole of the spleen is embolized, which are related to the restriction of breathing caused by the left upper quadrant pain after PSE, pleural reaction, and inadequate lymph drainage of inflammatory effusions. Mild and moderate pleural effusion might be absorbed after effective antibiotics and pain alleviation therapy, while thoracentesis should be performed for great pleural effusion. Selective embolization of the middle part or the lower pole of the spleen should reduce the incidence of these kinds of complications [25]. The interventional radiologist must be aware of pulmonary artery embolization after PSE, because it has resulted in death in one report [53].

### 8.3. Severe Infection

Severe complications of PSE include peritonitis and splenic abscess that are related to excessive embolic volume, damaged immune function of the spleen, poor aseptic processing during the operation [2], and retroinfection by intestinal anaerobic bacterium which may cause peritonitis. Antibiotics therapy is necessary for these conditions, while puncture-drainage or puncture-wash treatment even surgical intervention may be needed in some cases [54].

### 8.4. Damage of Renal Function

The administration of contrast media, redistribution of organ blood flow, and hypoperfusion of kidney are related to the damage of renal function after PSE. The pathogenesis of contrast nephropathy is closely related to a transient local cellular hypoxia response and altered intrarenal hemodynamics, rather than acute tubular injury [55]. Contrast media produce decrease in outer medullary blood flow (MBF) and oxygen tension, resulting in hypoperfusion and hypoxia. In most of the cases, the damage of renal function is reversible and can be prevented partially by some agents such as adenosine receptor antagonist [56].

### 8.5. Damage to Liver Function

In evaluating the damage to liver function after PSE, preoperative Child-Pugh grading has proven to be a useful method. Although necrosis in the embolic area, reducing in portal venous flow, portal vein thrombosis, and the administration of contrast media are all responsible for the liver damage after PSE [46, 47], liver function reserve is still the most important factor to be considered. More severe damage to liver function after PSE was found in patients with higher score of Child-Pugh grading before PSE. In patients with noncompensated cirrhosis, it must be kept in mind that death was reported from acute on chronic liver failure [54].

There are a lot of arguments about influence of liver function after PSE recently. The proponents suggest that cut of splenic arterial flow improves hepatic ischemia and reperfusion injury [57], and ameliorated the remnant liver function in and excessively hepatectomized rats [58]. On the other hand, when there is ischemia, the nuclear factor kappaB will be activated and can bind to special sequence in the promoters of budget genes, which can upregulate the expression of cytokines including TNF-alpha and ICAM-1 mRNA to result in ischemia reperfusion injury of the liver [59]. Such theories comprise complicated mechanisms and further studies are warranted.

### 8.6. Portal Vein Thrombosis

The decreased portal vein flow and the rapidly increased platelet count after excessive embolization may result in hypercoagulable state of the portal vein flow. It is related to the portal vein thrombosis and can be overcome by heparin administration [27, 44].

### 8.7. Other Rare Complications

Inadvertent embolization of the pancreatic artery and the administration of contrast media may cause pancreatitis after PSE [50, 51], but the condition can usually be relieved with expectant treatment. External injury or aggravating activities may result in splenic rupture [2] in those patients with abscesses or pseudocysts after PSE. In that case, surgical intervention should be performed immediately. Similar to that happened in the portal vein, the decreased splenic vein flow may cause thrombosis in this vein as well [44]. However, no severe consequences will be encountered if the thrombus locates only in the splenic vein, and repeated embolization of small embolic volumes each time may prevent this complication from happening [48].

## 9. Option of the Volume to Embolize 

The therapeutic effect and the complication occurrence of PSE to treat hypersplenism are tightly related to the infarcted size of the spleen. Opinions upon the optimal volume to embolize are still controversial, even though the documented volume of embolized spleen mass in percentage varied from 30% to 70%. However, some of the current opinions suggest that the volume of the first embolization should be less than 70% of the total spleen mass in order to minimize the incidence of complications [27, 48].

According to our practice, patients with hematological diseases related to hypersplenism deserve the biggest area of spleen embolization [60], while the percentage of the spleen to be occluded of cirrhosis patients with portal hypertension should be higher than that of the others, which may reduce the splenic vein return and relieve portal hypertension [27]. For liver cancer patients [43] with hypersplenism, who are supposed to accept interventional therapy repeatedly, multiple procedures of embolization and a modest area to be embolized each time should be recommended as better.

In children, PSE should be treated distinctively with caution. With more active metabolism, tissue repair in the embolized spleen in children should be faster than that in adults, so the embolized volume should be 10% more accordingly. In the study conducted by Watanabe et al. [61], the authors found that reembolization is not likely to be necessary in children patients who have had splenic embolization with an infarction rate more than 80%.

However, multiple embolization procedures with lower single infarction rate were suggested as an alteration choice as well. The infarction rate in first embolization should be about 20%–40% and about 20%–30% in the reembolization procedure 2 or 3 months later. The complications of this method are minor and less frequent, but the cost is higher and the hospitalization duration is prolonged as well [25].

## 10. Limitations of PSE

In the early times of PSE practice, serious complications and their grave consequences, such as splenic abscess, rupture of the spleen, pancreatitis, pneumonia, and septicemia, limited the wide use of this procedure [2, 5]. With improvement of the techniques, PSE by the injection of agents via a catheter comprising approximately 30–70% of the splenic parenchyma is now a safe method [4]. However, some points should be noted with concern when this procedure is practised.

### 10.1. Selection of the Site to Embolize

Although embolization of the periphery of the spleen (Figure 2) is the most popular technique of PSE [1, 2], it has been suggested [25] that embolization of the middle or lower part of the spleen (Figure 1) should reduce such complications as pneumonia, left upper quadrant abdominal pain, and pleural reaction on the left and be beneficial to evaluating the embolized volume as well. However, it is still argued that whether superselective catheterization is possible for the hypersplenism patients with splenic artery circuity and dilatation, and the long-term relapse rate of hypersplenism after this technique is still doubtful [26, 44].

### 10.2. Evaluation of Embolized Volume

Another limitation of PSE is the lack of a precise quantitative method for the evaluation of embolized volume during the procedure. The blood flow rate is traditionally used as an indicator; however, it is highly dependent on the experience of the operator. An objective method to quantitate the blood flow reduction during the procedure is still unavailable. The video dilution technique (VDT) has earlier been used to measure blood flow in regional arteries using video cassette replay. By adapting the VDT concept of relative flow to digital subtraction angiography, it is possible to calculate the flow reduction instantly following each injection of embolic material [62]. Mei et al. [63] found that in a given spleen volume, the number of artery branches of 1 mm diameter is positively correlated to the number of gelatin sponge pieces which should be used in PSE. Although it could be used as an indicator for evaluation of embolized volume of spleen, it still has many limitations in practice; for example, several randomly flowing particles may block the same artery branch, so that the ratio of particle to artery may be influenced [19].
